# Phylodynamic Characterization of an Ocular-Tropism Coxsackievirus A24 Variant

**DOI:** 10.1371/journal.pone.0160672

**Published:** 2016-08-16

**Authors:** Yung-Chang Yen, Pei-Huan Chu, Po-Liang Lu, Yung-Cheng Lin, Yong-Ying Shi, Li-Chiu Chou, Chu-Feng Wang, Yi-Ying Lin, Hui-Ju Su, Chien-Ching Lin, Jing-Yun Zeng, Yu-Chang Tyan, Guan-Ming Ke, Pei-Yu Chu

**Affiliations:** 1 Department of Ophthalmology, Chi Mei Medical Center, Liou-Ying, Tainan, Taiwan; 2 Department of Nursing, Min Hwei College of Health Care Management, Tainan, Taiwan; 3 Division of Cardiology, Department of Medicine, Wei Gong Memorial Hospital, Miaoli, Taiwan; 4 School of Medicine, College of Medicine, Kaohsiung Medical University, Kaohsiung, Taiwan; 5 Department of Laboratory Medicine, Kaohsiung Medical University Hospital, Kaohsiung, Taiwan; 6 Institute of Bioscience and Biotechnology, National Taiwan Ocean University, Keelung, Taiwan; 7 Department of Medical Laboratory Science and Biotechnology, College of Health Sciences, Kaohsiung Medical University, Kaohsiung, Taiwan; 8 Department of Medical Imaging and Radiological Sciences, Kaohsiung Medical University, Kaohsiung, Taiwan; 9 Graduate Institute of Medicine, College of Medicine, Kaohsiung Medical University, Kaohsiung, Taiwan; 10 Institute of Medical Science and Technology, National Sun Yat-sen University, Kaohsiung, Taiwan; 11 Center for Infectious Disease and Cancer Research, Kaohsiung Medical University, Kaohsiung, Taiwan; 12 Graduate Institute of Animal Vaccine Technology, College of Veterinary Medicine, National Pingtung University of Science and Technology, Neipu, Pingtung, Taiwan; Fudan University, CHINA

## Abstract

Recent phylodynamic studies have focused on using tree topology patterns to elucidate interactions among the epidemiological, evolutionary, and demographic characteristics of infectious agents. However, because studies of viral phylodynamics tend to focus on epidemic outbreaks, tree topology signatures of tissue-tropism pathogens might not be clearly identified. Therefore, this study used a novel Bayesian evolutionary approach to analyze the A24 variant of coxsackievirus (CV-A24v), an ocular-tropism agent of acute hemorrhagic conjunctivitis. Analyses of the 915-nucleotide VP1 and 690-nt 3D^pol^ regions of 21 strains isolated in Taiwan and worldwide during 1985–2010 revealed a clear chronological trend in both the VP1 and 3D^pol^ phylogenetic trees: the emergence of a single dominant cluster in each outbreak. The VP1 sequences included three genotypes: GI (prototype), GIII (isolated 1985–1999), and GIV (isolated after 2000); no VP1 sequences from GII strains have been deposited in GenBank. Another five genotypes identified in the 3D^pol^ region had support values >0.9. Geographic and demographic transitions among CV-A24v clusters were clearly identified by Bayes algorithm. The transmission route was mapped from India to China and then to Taiwan, and each prevalent viral population declined before new clusters emerged. Notably, the VP1 and 3D^pol^ genes had high nucleotide sequence similarities (94.1% and 95.2%, respectively). The lack of co-circulating lineages and narrow tissue tropism affected the CV-A24v gene pool.

## Introduction

The coxsackievirus A24 variant (CV-A24v), an enterovirus (EV) C species of Picornaviridae, is an antigenic mutant of CV-A24. An ocular tropism results from the use of a sialylated glycoprotein as a cellular receptor [1], and CV-A24v is known to be a major causative agent of acute hemorrhagic conjunctivitis (AHC) with pandemic potential [2]. After the first report of an AHC outbreak caused by CV-A24v in Taiwan in 1985–1986, CV-A24v intermittently reappeared in 1988–1989, 1990–1991, 1994, 2000–2002, 2006–2007 and 2010. Four genotypes (I–IV, GI–GIV) were depicted in our previous report [3, 4]. Epidemic episodes of CV-A24v can be divided into four periods. In the *emergence* period during the 1970s, CV-A24v was limited to Asia. The *geographic expansion* period in the 1980s was characterized by outbreaks in Asia, Central America, and North Africa and was followed by a *quiescence* period, which lasted a decade. Finally, CV-A24v re-emerged in the *wide dispersal period* after 2000 [3]. Since CV-A24v outbreaks occur periodically in tropical areas and involve large populations, the emergence of CV-A24v is a major public health concern, particularly in densely populated areas. Additional contributors to EV transmission are global warming, globalization, and high population densities. The most at-risk age group has shifted from adults (before 1999) to teenagers (after 2000) [5, 6], possibly due to technological changes such as sharing computing devices by multiple users.

Incorporating data types in addition to viral sequences in a Bayesian phylodynamic analysis can reveal interactions among epidemic episodes, evolution, selection, and demographic and spatiotemporal characteristics of transmission [7]. For example, in a Bayesian phylogenetic tree, a ladder-like trunk and short terminal branches indicate continual immune-driven selection and acute infection [8]. However, most EV studies have focused on pathogens causing severe systemic disease rather than on pathogens with narrow tissue tropism. Hypothetically, if the evolution of a virus results in the preferential targeting of specific tissues, special growth environments may limit variation in this virus. To investigate the phylodynamic characteristics of this ocular tropism pathogen, this study analyzed the sequences of serotype-specific VP1 and species-specific 3Dpol regions from CV-A24v. In each region, molecular characteristics were identified by variation detection. Time-scaled phylogenies inferred with a Bayesian Markov chain Monte Carlo (BMCMC) sampling framework were used to elucidate the phylodynamic properties and spatiotemporal transmission history of CV-A24v.

## Materials and Methods

### Ethics statement

This study randomly selected 21 strains from the CV-A24v-positive viral strains isolated during the 1985–2010 outbreaks in Taiwan by Kaohsiung Medical University Hospital and by the Taiwan Centers for Disease Control, Taiwan. All samples were de-identified and analyzed anonymously. This study was approved by the ethics committees of Kaohsiung Medical University Hospital. Informed consent was waived because experiments were performed on viral isolates obtained in routine clinical procedures and without accessing patient medical data.

### Specimens, viral RNA extraction, reverse transcription (RT)-polymerase chain reaction (PCR), and sequencing

All CV-A24v strains were isolated from corneal swabs. The RNA extraction, RT-PCR and sequencing procedures were performed as described previously [9] except for the use of PCR primers (Table 1). Primer set designed for the 3D^pol^ region of CV-B2, a serotype belongs to species EV-B, in our previous study was used for amplification in this study [10]. Briefly, viral RNA was extracted using a QIAmp viral RNA purification kit (Qiagen, Chatsworth, CA, USA). The QIAGEN one-step RT-PCR kit was used to perform RT-PCR in a single tube. The ABI Prism Ready Reaction Dideoxy Terminator cycle sequencing kit (Model 3730, version 3.4, Applied Biosystems, Foster City, CA) was used for cycle sequencing of purified PCR products. Sequence data obtained for the 915-nucleotide (nt, 2491–3405) VP1 and 690-nt (6490–7179) 3Dpol genes were submitted to GenBank under accession numbers AB901473–AB901493 and AB908967–AB908987, respectively.

**Table 1:** Primer sets used for coxsackievirus A24 variant.

Gene	Primer^a^	Sequence	Position^b^	Reference
VP3	PY-06-VP3F	GCATTAAAGATGACTTTACAG	2303–2323	This study
VP1	222R	CICCIGGIGGIAYRWACAT	2820–2834	[11]
VP1	292F	MIGCIGYIGARACNGG	2627–2639	[11]
2A	PY-07-2AR	GATAATTGCAGATCTTGTA	3448–3467	This study
3D^pol^	PY-03F	GTYACMTATGTGAARGATG	6472–6490	[10]
3D^pol^	PY-04R	CTTCATTGGCATTACTGGATG	7173–7194	[10]

^a^F: Forward primer, R: reverse primer.

^b^Numbering system used for the coxsackievirus A24v strain (Accession No. D90457).

### Sequence alignment and variation detection

After excluding sequences with nonsense or frameshift mutation, all sequences with a full length VP1 or 3D^pol^ region from GenBank were stratified by isolation year and location. The final analysis datasets included 111 sequences of VP1 and 44 sequences of 3D^pol^ (S1 Table). Each dataset included 21 Taiwan strains and the prototype strain EH24/70. The Mafft.v7 program [12] was used for multiple sequence alignment. Conserved residues in deduced amino acid (aa) sequences were graphically visualized by WebLogo [13]. Series variations were characterized as described previously [14]. Briefly, RDP3.44 [15], DAMBE5.2 [16], and the MEGA6[17] programs were used to analyze recombination, site-specific variability, and pairwise comparisons, respectively, and DataMonkey website [18] was used for selection detection. To understand how variations caused tissue tropism changes, a further VP1-deduced aa dataset of 25 strains of CV-A24 were compared with CV-A24v in WebLogo. This dataset included all 915 nt full length CV-A24 strains in GenBank.

### Phylogenetic and phylodynamic analyses

The outgroup included prototype strains of CV-A24 and poliovirus (PV) types 1–3. Two datasets, with and without the outgroup, were analyzed in both the VP1 and 3D^pol^ regions. The Bayesian Evolutionary Analysis Sampling Tree (BEAST) program (v.1.8.2) was used for phylodynamic inference [19], and the MEGA6 program was used for the neighbor-joining (NJ) and maximum likelihood (ML) evolutionary tree algorithms [20]. The models with the best fit to the NJ and ML trees were identified by comparing the Akaike information criterion model (AICM) [21] values via jModeltest 2.4.1. Bayesian analyses were run for 30 million states, and sampling was performed once every 30,000 generations until the distributions were found stationary. The best-fit model composition was determined by comparing the AICM values via Trace v1.6.

The strongest geographical links were then identified usinga standard discrete phylogeographic analysis. The BEAST program was used to co-estimate nt substitution rates, the mean time to most recent common ancestor (TMRCA), and to reconstruct the viral population and spatiotemporal dynamic history for the dataset without the outgroup. Estimated parameters with ESS > 200 were considered reliable. All estimation parameters are presented as means and 95% highest posterior densities (HPDs). The FigTree v.1.4.2 program was used to construct and visualize the maximum clade credibility tree. A supported node was defined as node with a posterior probability (PP) > 0.90 in the maximum clade credibility (MCC) tree or a node with bootstrap (BS) > 70% (1000 replicates) in the NJ and ML trees. The SPREADv1.0.6 program [22] was used to estimate transition rates between discrete geographic areas. In Bayes factor (BF) tests for significant non-zero rates of CV-A24v dispersal, a reliable BF value was defined as >3. Finally, ArcGIS explorer (Environmental Systems Research Institute, Redlands, CA, USA) was used to visualize the major routes of transmission between two discrete geographic areas.

## Results

### Sequence variation in the VP1 and 3D^pol^ regions

The VP1 and 3D^pol^ regions revealed high similarity and no recombination events. For example, the maximum differences in nt/aa sequences of the VP1 and 3D^pol^ regions among the CA24v strains were 85.4%/94.1% and 85.5%/95.2%, respectively. The high similarity might have resulted from gene variation being limited by narrow-tissue-tropism. The single-likelihood ancestor counting analysis revealed negative selection in both regions with dN/dS values of 0.087 and 0.035, respectively. For the VP1 and 3D^pol^ regions, the model composition with the best fit (i.e. lowest AICM value) was the Shapiro-Rambaut-Drummond-2006, uncorrelated exponential relaxed, Bayesian skyline plot (BSP) model (S2 Table). The estimated substitution rates were 7.40×10^−3^ (95% HPD: 6.08×10^−3^–8.68×10^−3^) substitutions/site/year (s/s/y) for VP1 and 6.07×10^−3^ (3.97×10^−3^–8.24×10^−3^) s/s/y for 3D^pol^.

**Fig 1:**
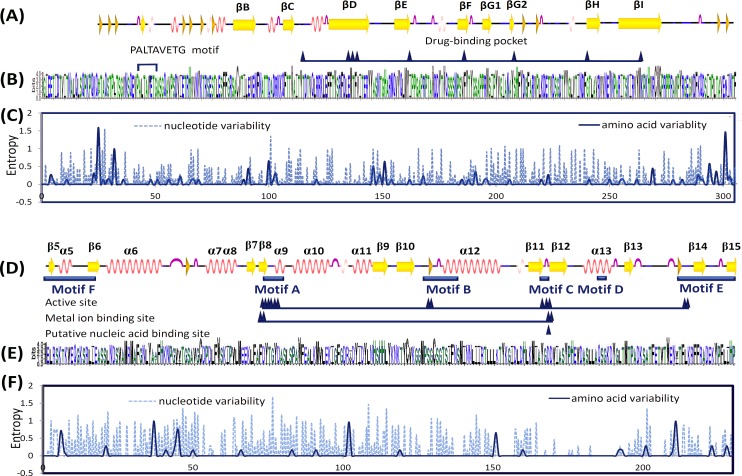
**Comparison of coxsackievirus A24v strains in (A–C) 305 codon sites of 111 VP1 sequences and in (D–F) 230 codon sites of 44 3Dpol sequences.** (A, D) Secondary structure guide (PDB ID codes 4Q4V for VP1, 2IJD for 3Dpol products). (B, E) Consensus residues graphically depicted by Weblogo. (C, F) Entropy-based variability plotted by DEMBA.

Fig 1A shows that the VP1 protein, which is a component of the viral capsid, contained a hydrophobic motif, 42-PALTAVETG-50, that might play a role in uncoating or cell entry [23], followed by an eight-strand β-barrel (βB–βI) and loops between the β-strands. Fig 1B and 1C show that the sites with the highest variability were the N-terminus, the C-terminus, and the outermost BC and EF loops. The CV-A24v aa sequences were highly conserved in comparison with CV-A24 aa sequences deduced by WebLog (S1 Fig). The CV-A24v revealed three variation sites: alanine (A) at the 111^th^ position, lucine (L) at the 159^th^ position, and valine (V) at the 224^th^ position. The corresponding positions in CV-A24 were proline (P); isoleucine (I), and threonine (T) or A. Most residues were conserved at the same sites in CV-A24 and CV-A24v. However, some common residues had different frequencies. Interestingly, most sites of variations were in the BC-loop, the N-terminus, and the C-terminus.

In the 3D^pol^ protein, which is an RNA-dependent RNA polymerase (RdRP), all polymerase structures had canonical right-hand folds with finger, palm, and thumb domains. The catalytic palm (A–D motif) subdomain was the most conserved region in most other polymerases whereas the finger and thumb domains showed structural differences in RdRp [24]. The partial 3D^pol^ gene analyzed in this study encompassed residues 161–390, which enclosed motifs A–F (Fig 1D). Non-synonymous mutation was sparse in non-β strand regions (Fig 1E and 1F).

### Phylodynamic analysis of CV-A24v based on VP1

**Fig 2:**
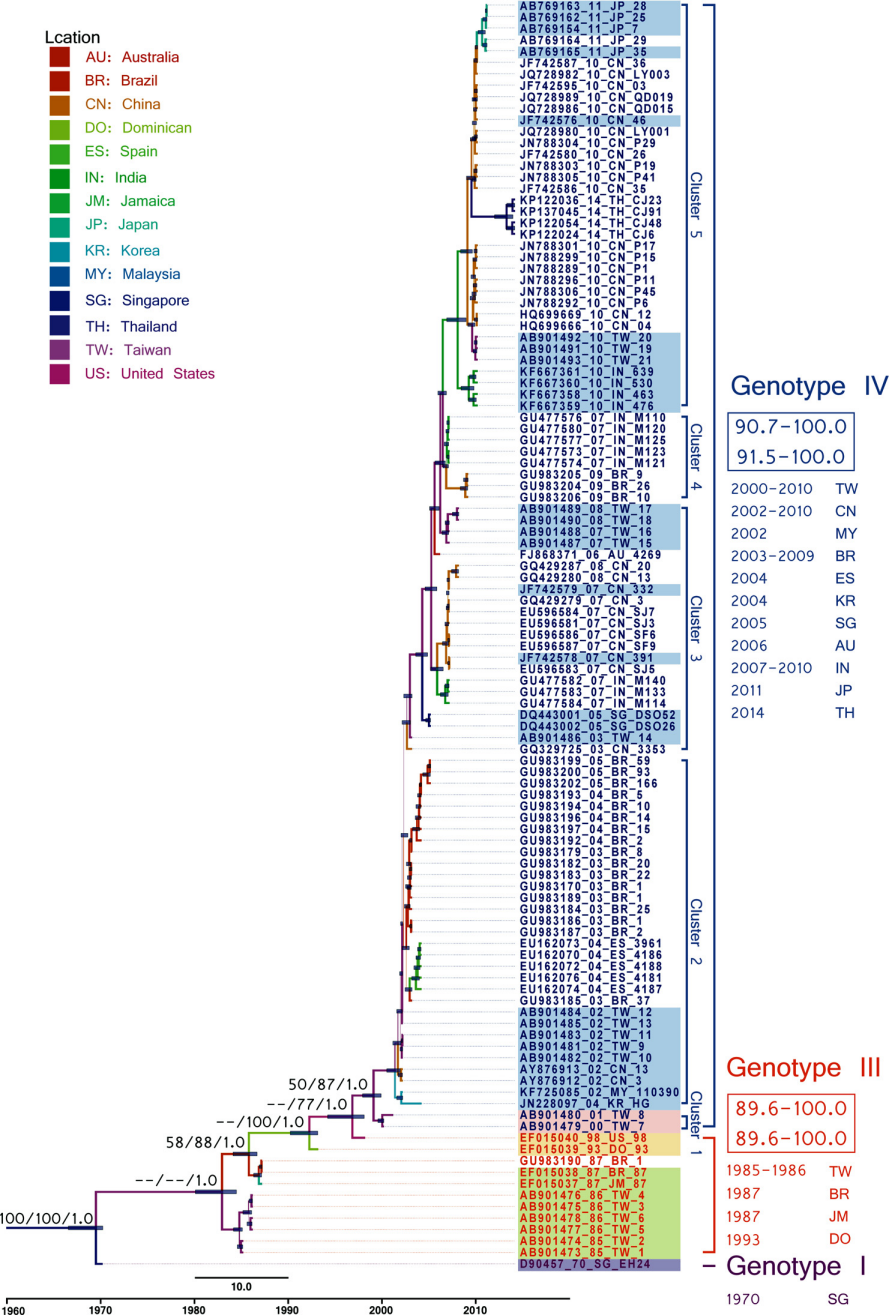
Maximum clade credibility phylogeny of 111 VP1 sequences of coxsackievirus A24v without outgroup. Blue bars at nodes indicate 95% HPDs of TMRCAs. Branch color indicates the likely location, and branch thickness indicates posterior probability from Bayesian inference (PP-BMCMC). Support values are also shown for major nodes and are indicated as BS-NJ/BS-ML/PP-BMCMC, where BS is the bootstrap value, NJ indicates the neighbor-joining method, and ML indicates the maximum likelihood method. The VP1 genotypes are shown on the right and are distinguished by color. Within-genotype nucleotide and amino acid similarities are also shown on the right. For comparison, the 3D^pol^ genotypes are distinguished by shading (GA: purple, GB: green, GC: yellow, GD: orange, and GE: blue). The branch length indicates the evolution time, and the scale bar at the bottom indicates the calendar time.

In both the VP1 and 3D^pol^ regions, phylogenetic analyses with and without the outgroup revealed similar topologies in NJ, ML and BMCMC trees. Strains in both regions tended to cluster temporally rather than spatially. The VP1 trees tended to be unbalanced with all strains chronologically clustered along a ladder-like backbone with short terminal branches (Fig 2 and S2A Fig). Short terminal clusters were interpreted as sequential extinction of dominant lineages and replacement by newer selection escape lineages. Most clusters circulated for 1–3 years (short terminal branches) before the backbone extended to subsequent clusters. Notably, backbones near the root (i.e., interior backbones) tended to be longer than backbones near the tip, especially after 2000, which indicated increasing nt similarity among evolving sublineages, possibly because the sequences stabilized as adaptive fitness increased.

Because early genotyping reports focused on the 3C region, the number of full VP1 sequences available from GenBank was insufficient for complete analysis, especially for VP1 sequences isolated before 2000. Although we previously identified four genotypes [3], no VP1 sequences for GII (isolated 1975–1976) have been deposited in GenBank. An extrapolated genotype demarcation of 10.4% depicted GI (prototype), GIII (isolated 1985–1998), and GIV (isolated after 1999) with supported values in all three methods. GIV was further depicted as five sequential clusters (C1–C5) [25, 26]. In the GIV backbone, series nodes between C2 and C3 had low support values. The GI cluster only included the prototype EH70 strain isolated in Singapore in 1970. Genotype III included strains isolated in Taiwan (1985–1986), Brazil and Jamaica (1987), Dominica (1993), and the USA (1998). GIV-C1 included strains isolated in Taiwan (2000–2001). The GIV-C2 strains had been isolated in a broad area that included China, Malaysia, and Taiwan (2002); Korea and Spain (2004); and Brazil (2003–2005). GIV-C3 included strains isolated in Singapore, China, India, and Taiwan (2003–2008) and one isolated in Australia (2006). GIV-C4 included strains isolated in India (2007) and Brazil (2009), whereas GIV-C5 included strains isolated in India, Taiwan, and China (2010); Japan (2011); and Thailand (2014). The discrete phylodynamic analysis revealed only two epidemiological linkage routes in GIV-C4 (2010) with BF > 3: the transmission from India to China (BF: 8.92) was followed by a transmission from China to Taiwan (BF: 4.2) (S1 Movie).

**Fig 3:**
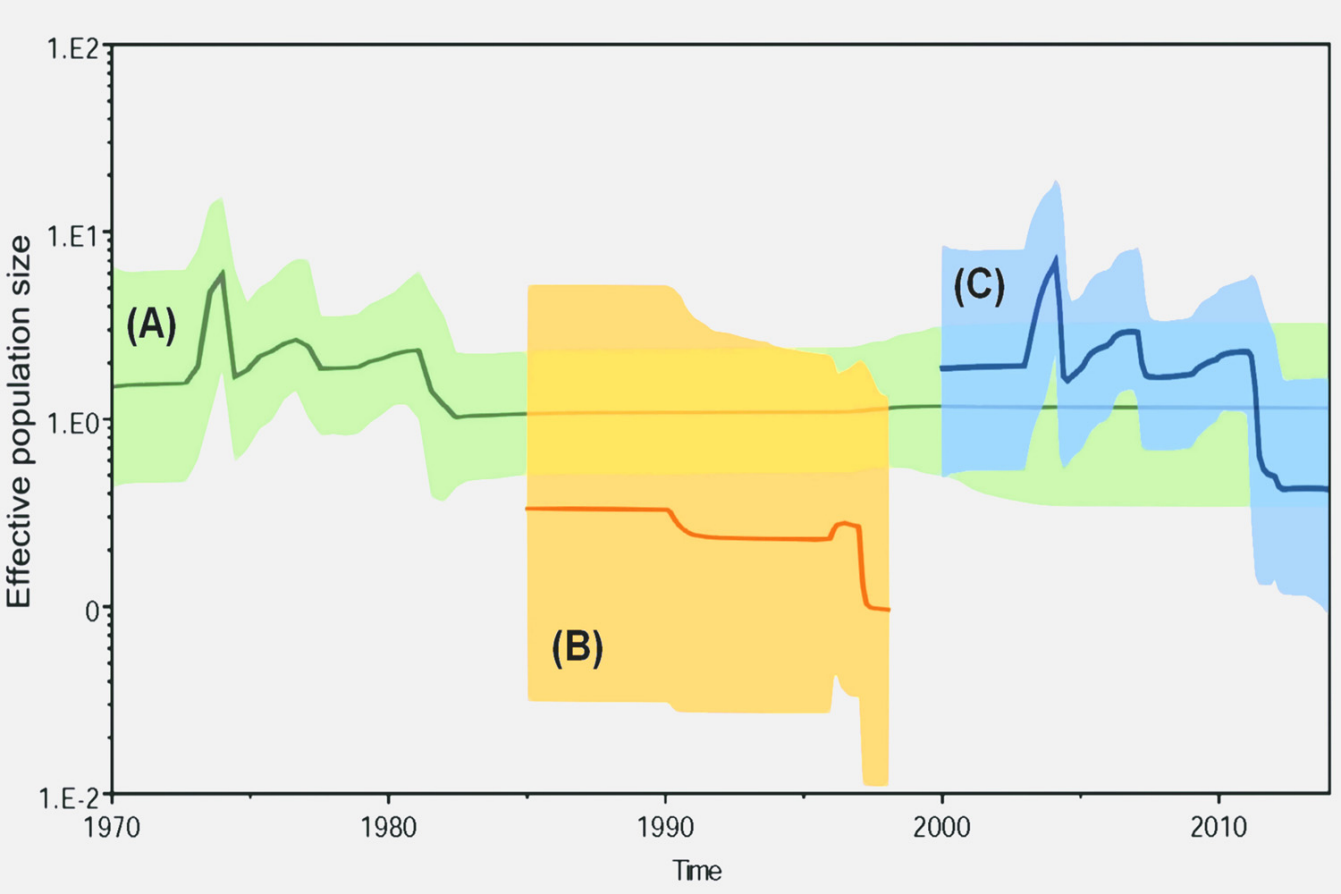
**Bayesian skyline plots of (A) 111 VP1 sequences, (B) 11 VP1 sequences of genotype III strains, and (C) 99 VP1 sequences genotype IV strains.** The x-axis is the time scale (years), and the y-axis is the logarithmic Neτ scale (where Ne is the effective population size and τ is the generation time). The thick solid line indicates the median estimates, and the shaded area indicates the 95% highest posterior density.

The BSP results support our previous suggestion that the global epidemiology of CV-A24v can be divided into four major stages: emergence and limited transmission in the 1970s (initial plateau), global transmission in the 1980s (three continuous waves), silence in the 1990s (steady decrease), and, finally, reemergence in the current stage (slight increase) (Fig 3). The BSP for GIII showed a consistently low effective population size of 0.33 followed by a drop after 1997. In contrast, the BSP for GIV revealed a high effective population size (1.9) followed by peaks in 2003, 2006, and 2010 and then a drop after 2014. Interestingly, the BSPs for the two genotypes showed no overlap. The estimated T_MRCA_ (95% HPD) was 1969 (1966–1970). For GIII and GIV, the estimated T_MRCA_ were 1984.8 (1984.5–1985.0) and 1999.5 (1998.0–2000.0), respectively. These data suggest a consistently high potential for CV-A24v outbreaks as the virus evolves.

CV-A24v has repeatedly caused AHC outbreaks in Taiwan since 1985. Each CV-A24v lineage remained prevalent for 1–2 years before being replaced by a newly emerging lineage. In addition, prevalent lineages clustered with strains isolated worldwide rather than with endemic strains. Examples in Taiwan include the GIII strains prevalent in 1985–1986 and the GIV-C2 strains prevalent in 2002. Strains isolated in 2003 and 2007 were clustered in two different sublineages of GIV-C3, and strains isolated in 2010 were clustered in GIV-C5. Since Taiwan is an island nation, these results suggest that each outbreak was caused by a newly emerging lineage that had been imported to Taiwan rather than by endemic strains that evolved locally.

#### Phylodynamic analysis of CV-A24v based on the 3D^pol^ region

**Fig 4:**
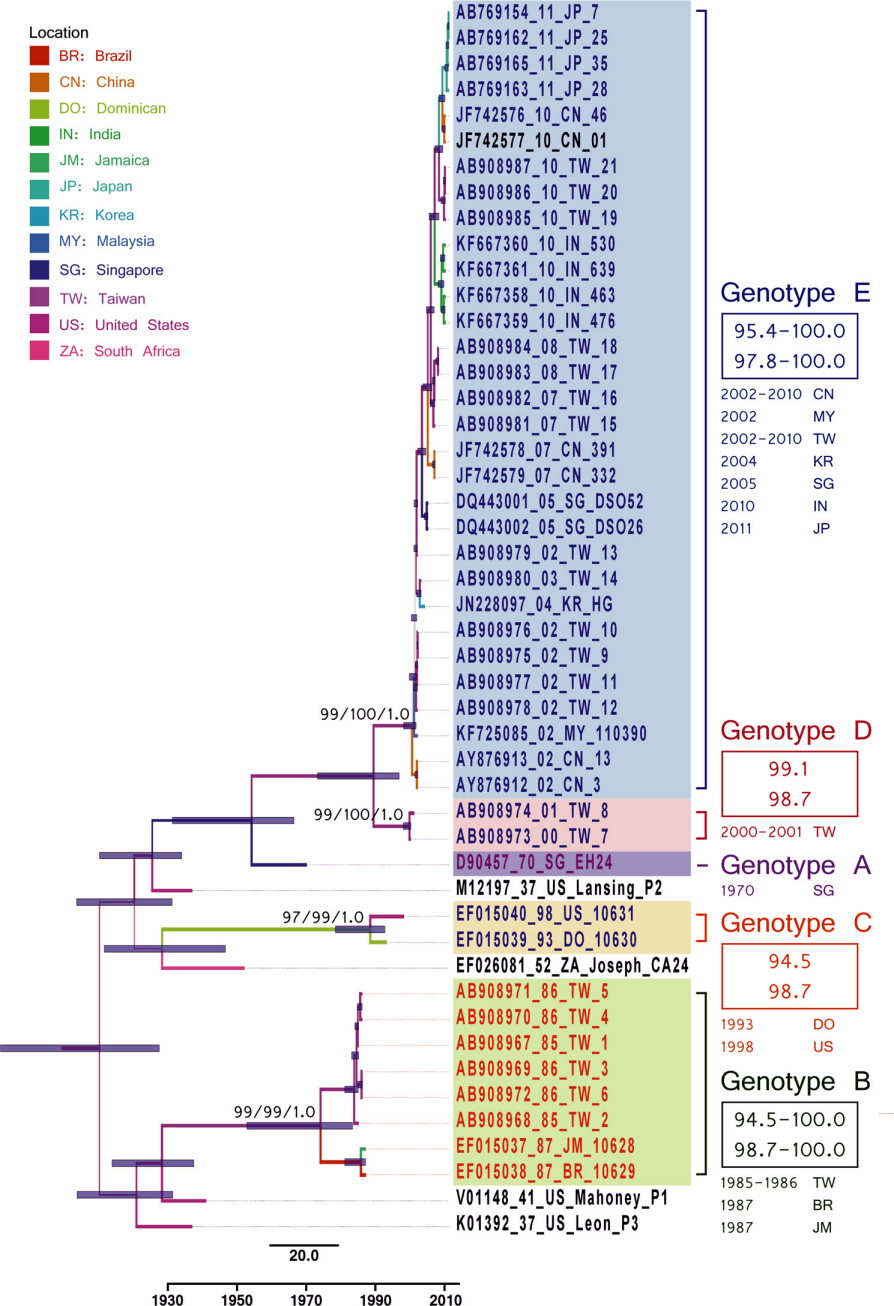
Maximum clade credibility phylogeny of 111 VP1 sequences of coxsackievirus A24v with outgroups. Branch thickness indicates support value (posterior probability, PP), and branch color indicates the most probable location. Support values for major nodes are also shown. The right side of the figure shows within-genotype nucleotide/amino acid similarities. The VP1 genotypes for each strain are distinguished by color (Genotype I: purple, Genotype III: orange, and Genotype IV: blue). The 3D^pol^ genotypes for each strain are distinguished by shading. Evolution time is indicated by branch length (see scale bar).

Whereas VP1 sequences of EVs are monophyletic by both species and serotype, 3D^pol^ sequences of EVs are monophyletic by species but polyphyletic by serotype (Fig 4, S2B Fig). Although all CV-A24v strains and the outgroup clustered together with high support values, each prototype strain appeared individually in a separate branch and with a low support value. The 3D^pol^ genotypes defined in this study were sequentially designated genotypes A–E. The prototype strain EH24 (isolated in 1970) was designated GA. The GB (eight strains isolated during 1985–1987) and GC (two strains isolated in 1993 and 1998) were both coordinates of GIII in the VP1 tree. The two GD strains (isolated during 2000–2001) and 31 GE strains (isolated after 2002) were both coordinates of GIII in the VP1 tree (S1 Table). Most 3D^pol^-based CV-A24v groupings were congruent with the VP1 grouping, i.e., the prototype appeared alone in a separate branch, GB and GC were congruent with GIII in VP1, and GD and GE were congruent with GIV in VP1. The VP1 tree showed high support values for a monophyletic group comprising all genotypes in a phylogenetic backbone. Intriguingly, the prototype strain of the CV-A24v (EH70, 1970) had a higher 3D^pol^ sequence similarity with the PV2 prototype strain than with the CV-A24 prototype strain, whereas the GB and GC strains (1985–1998) resembled the PV1 prototype strain. Subsequently isolated strains (GD and GE strains isolated after 1999) again resembled the prototype PV2 strain (Fig 4, S3 Table), which indicated that the recombination events between the VP1 and 3D^pol^ regions might have occurred when GA evolved to GB and when GC evolved to GD. Moreover, several clusters isolated during 2000–2004 with low support values in GIV of the VP1 tree also had low support values in GE of the 3D tree.

## Discussion

Studies of EV define the term ‘genotype’ as a cluster of genetically related viruses with less than 15% nt divergence [27]. However, this definition is clearly inadequate for CV-A24v due to the high similarity among VP1 sequences. Reported mutation rates for CV-A24v range from 6.15 to 7.86×10^−3^ s/s/y [28, 29], and those for EV range from 3.40×10^−3^ to 1.19×10^−2^ s/s/y for VP1 and 5.53×10^−3^ to 1.17×10^−2^ s/s/y for 3D^pol^ [30]. The CV-A24v isolates analyzed herein also exhibited high sequence similarity with those recently reported in the literature [28, 29], which indicates that sequences for tissue-specific pathogens such as CV-A24v may be fixed by adaptation. The CV-A24 and CV-A24v revealed high level of sequence similarity. Notably, however, GenBank contained only 25 full VP1 sequences of CV-A24. The sites of variation were concentrated in the outermost BC-loop. The conformational rigidity of proline affected the 111^th^ residue in the secondary structure of CV-A24 and might have had an important role in tissue tropism.

Most previous reports used a neighbor-joining algorithm for analyses based on the 3C region [3, 4, 26, 31, 32]. Genotypes I–II included early strains isolated in Asia in 1975 [33]. Genotype III strains appeared in 1985 and spread from Asia to Africa and France [4]. After a silent period in the 1990s, GIV emerged in 2000 [3]. The last reported outbreak was in Thailand in 2014 [29]. Five subgenotypes of GIV have been identified as cluster 1–5 (C1–C5) [3, 26]. The BSP for GIV revealed three waves with peaks in 2003, 2006, and 2010, which may indicate the emergence of GIV-C2, GIV-C3, and GIV-C5, respectively. Although this study is limited by insufficient full VP1 sequences in GenBank, the phylogeny was a typical unbalanced topology with a ladder-like backbone extending to the tip of the tree with high support values on the major backbone. Another notable feature was multiple lineages co-circulating simultaneously, which is common in other EVs but apparently not in CV-A24v.

High recombination rates are often reported in EV [34], especially in EV-C. Since the recombination rate is proportional to the nt distance and 3D^pol^ is located at the farthest C-terminal end of the viral open reading frame, the phylogenetic incongruence between VP1 and 3D^pol^ is widely used as an indicator of recombination within these two gene regions [35]. This study revealed no recombination events, which are rarely reported in CV-A24v. Phenomena such as one lineage per outbreak and high gene similarity between prevalent strains might hinder detection of viral recombination.

In addition to the 3D^pol^ sequences for the prototype PV2-like strains, which switched to a PV1-like strain (GB) and then back to PV2-like strains (GD and GE), the low support values for strains isolated during 2002–2005 in both the VP1 and 3D^pol^ trees suggest that the dormant period of CV-A24v ended in the 1990s and that CV-A24v is now in the reemergence and worldwide transmission stage. The bottleneck effect and founder effect in a virus with narrow tissue tropism can drastically reduce the original CV-A24v gene pool.

## Conclusion

To achieve a clear depiction of the evolution of CV-A24v, we performed VP1- and 3D^pol^-based analyses in this ocular-tropism virus and reconstructed its phylodynamic history. In both the VP1 and 3D^pol^ regions, low sequence variation and the absence of recombination suggest that narrow tissue tropism may limit sequence variation. A chronological trend clearly revealed by the VP1 dendrogram obtained by BMCMC is the occurrence of outbreaks as novel dominant clusters emerge. Specifically, CV-A24v outbreaks resulted from newly emerging clusters that circulated for 1–2 years before being replaced by subsequently emerging subclusters. Notably, the T_MRCA_ estimates obtained in this study indicate that CV-A24v has high potential for outbreaks because outbreaks of the prevalent lineage occur soon after it evolves. The prevalent lineage is then trimmed by herd immunity. The evolve-outbreak-trimming cycle results in a phylogenetic tree with an imbalanced topology. Thus, two characteristics of CV-A24v limited its gene pool: a narrow tissue tropism and outbreaks caused by single prevalent lineage.

## Supporting Information

S1 Fig Comparison of consensus amino acid residues in VP1 of CV-A24v and CV-A24. Consensus residues were depicted by Weblogo. Red arrows in the above secondary structure guide (PDB ID codes 4Q4V) indicate positions at which residues differed. Blue arrows indicate positions at which residues were at the same sites but had different frequencies.
(TIF)

S2 Fig Maximum clade credibility phylogenies of CV-A24v. (A) 111 VP1 sequences with outgroup. (B) 44 3D^pol^ sequences without outgroup. For each branch, the thickness indicates the support values (PP), and the color indicates the most probable location. Support values are also given for major nodes. The genotypes and nt/aa similarities within genotypes are shown on the right. For each strain, the VP1 genotypes are differentiated by color (Genotype I: purple, Genotype III: orange, and Genotype IV: blue) whereas the 3Dpol genotypes are differentiated by shading (GA: purple, GB: green, GC: yellow, GD: orange, and GE: blue). The branch length is proportional to the evolution time, and the scale bar is proportional to calendar time.
(TIF)

S1 Movie Transmission of CV-A24v. Pushpins show the locations of sampling sites, and the circle sizes indicate the number of lineages identified in the location during the given period. The lines between locations indicate transmission routes, and the opacity indicates the support value of the node.
(MP4)

S1 Table List of sampled CV-A24v strains. (PDF)

S2 Table Best models according to Akaike's information criterion (AICM). (PDF)

S3 Table Pairwise comparison of 3D^pol^ nucleotide and amino acid similarity between each CV-A24v genotype and prototype strains of CV-A24 and poliovirus 1–3. (PDF)
