# Supplementary figures and images for: Phylodynamic Characterization of an Ocular-Tropism Coxsackievirus A24 Variant
Source: PLoS One. 2016 Aug 16;11(8):e0160672. doi: 10.1371/journal.pone.0160672 (PMC4987047; doi:10.1371/journal.pone.0160672)

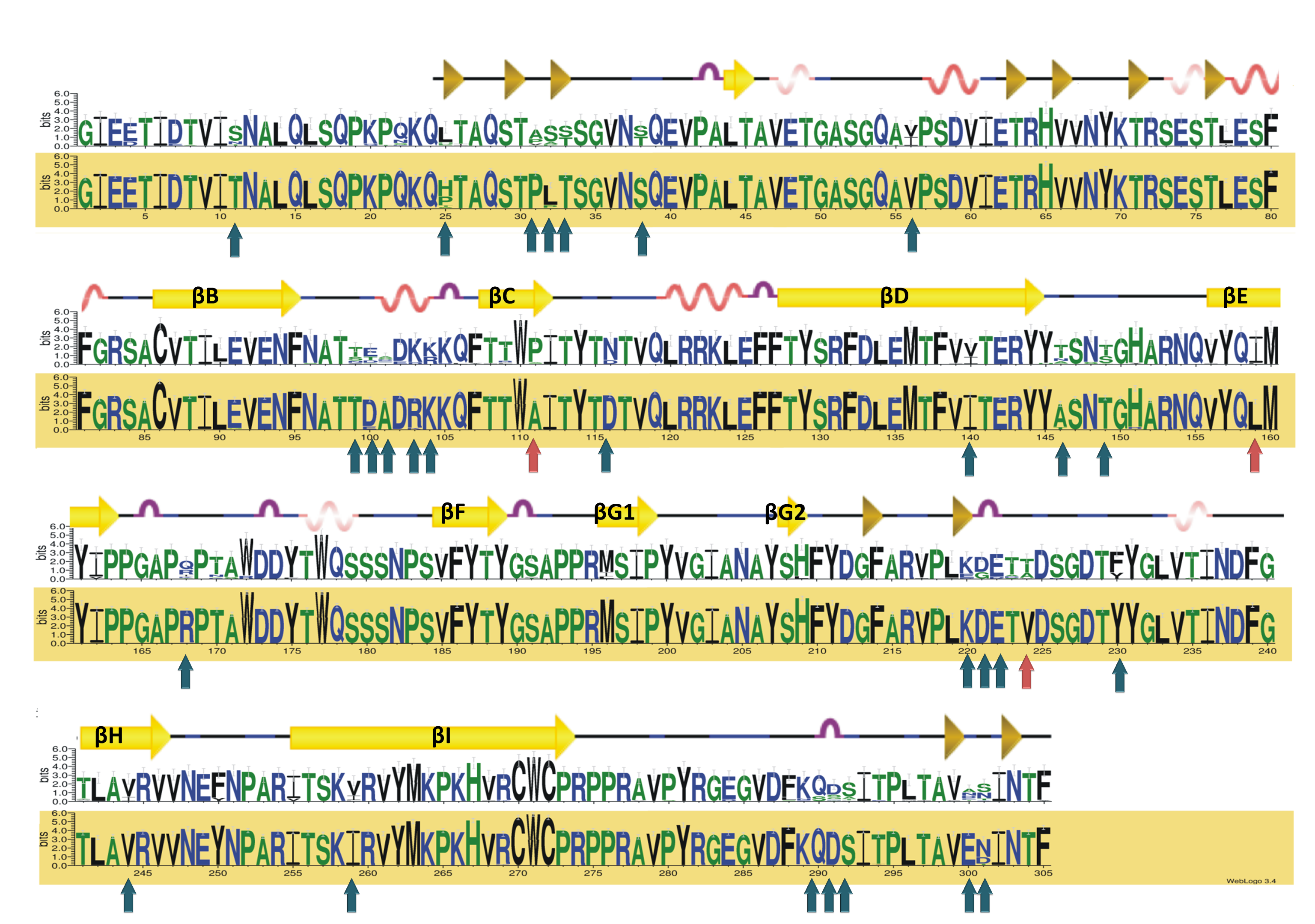

Supplement: S1 Fig — Consensus residues were depicted by Weblogo. Red arrows in the above secondary structure guide (PDB ID codes 4Q4V) indicate positions at which residues differed. Blue arrows indicate positions at which residues were at the same sites but had different frequencies. (TIF) [file pone.0160672.s001.tif]

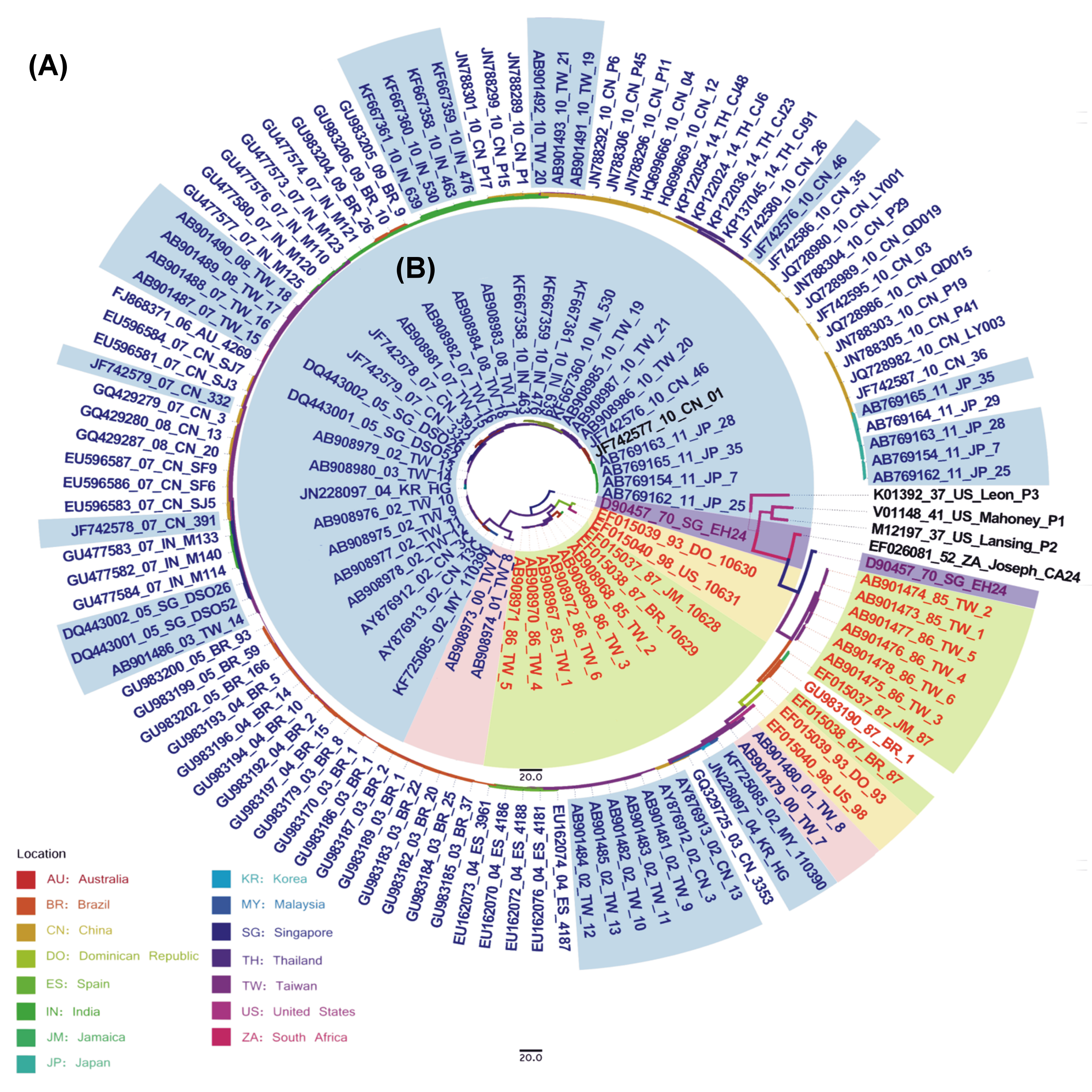

Supplement: S2 Fig — (A) 111 VP1 sequences with outgroup. (B) 44 3Dpol sequences without outgroup. For each branch, the thickness indicates the support values (PP), and the color indicates the most probable location. Support values are also given for major nodes. The genotypes and nt/aa similarities within genotypes are shown on the right. For each strain, the VP1 genotypes are differentiated by color (Genotype I: purple, Genotype III: orange, and Genotype IV: blue) whereas the 3Dpol genotypes are differentiated by shading (GA: purple, GB: green, GC: yellow, GD: orange, and GE: blue). The branch length is proportional to the evolution time, and the scale bar is proportional to calendar time. (TIF) [file pone.0160672.s002.tif]
