# Supplementary material for: Phylodynamic Characterization of an Ocular-Tropism Coxsackievirus A24 Variant
Source: PLoS One. 2016 Aug 16;11(8):e0160672. doi: 10.1371/journal.pone.0160672 (PMC4987047; doi:10.1371/journal.pone.0160672)
Supplement: S1 Table — (PDF) [file pone.0160672.s004.pdf]

S1 Table. List of sampled CV-A24v strains.

(A) The CV-A24v strains isolated in this study

Assigned name	Isolation yr.	Isolation location	VP1 accession no.	3D accession no.
1	1985	Taiwan	AB901473	AB908967
2	1985	Taiwan	AB901474	AB908968
3	1986	Taiwan	AB901475	AB908969
4	1986	Taiwan	AB901476	AB908970
5	1986	Taiwan	AB901477	AB908971
6	1986	Taiwan	AB901478	AB908972
7	2000	Taiwan	AB901479	AB908973
8	2001	Taiwan	AB901480	AB908974
9	2002	Taiwan	AB901481	AB908975
10	2002	Taiwan	AB901482	AB908976
11	2002	Taiwan	AB901483	AB908977
12	2002	Taiwan	AB901484	AB908978
13	2002	Taiwan	AB901485	AB908979
14	2003	Taiwan	AB901486	AB908980
15	2007	Taiwan	AB901487	AB908981
16	2007	Taiwan	AB901488	AB908982
17	2008	Taiwan	AB901489	AB908983
18	2008	Taiwan	AB901490	AB908984
19	2010	Taiwan	AB901491	AB908985
20	2010	Taiwan	AB901492	AB908986
21	2010	Taiwan	AB901493	AB908987

(B) Reference CV-A24v strains isolated worldwide

Assigned name	Isolation yr.	Isolation location	VP1 accession no.	3D accession no.
EH24	1970	Singapore	D90457	D90457
7	2011	Japan	AB769154	AB769154
25	2011	Japan	AB769162	AB769162
28	2011	Japan	AB769163	AB769163
29	2011	Japan	AB769164	AB769164
35	2011	Japan	AB769165	AB769165
3	2002	China	AY876912	AY876912
13	2002	China	AY876913	AY876913
DSO52	2005	Singapore	DQ443001	DQ443001

DSO26	2005	Singapore	DQ443002	DQ443002
87	1987	Jamaica	EF015037	EF015037
87	1987	Brazil	EF015038	EF015038
93	1993	Dominican	EF015039	EF015039
98	1998	United States	EF015040	EF015040
HG	2004	Korea	JN228097	JN228097
463	2010	India	KF667358	KF667358
476	2010	India	KF667359	KF667359
530	2010	India	KF667360	KF667360
639	2010	India	KF667361	KF667361
110390	2002	Malaysia	KF725085	KF725085
46	2010	China	JF742576	JF742576
391	2007	China	JF742578	JF742578
332	2007	China	JF742579	JF742579
01	2010	China	X	JF742577
4269	2006	Australia	FJ868371	X
1	2003	Brazil	GU983170	X
8	2003	Brazil	GU983179	X
20	2003	Brazil	GU983182	X
22	2003	Brazil	GU983183	X
25	2003	Brazil	GU983184	X
37	2003	Brazil	GU983185	X
1	2003	Brazil	GU983186	X
2	2003	Brazil	GU983187	X
1	2003	Brazil	GU983189	X
1	1987	Brazil	GU983190	X
2	2004	Brazil	GU983192	X
5	2004	Brazil	GU983193	X
10	2004	Brazil	GU983194	X
14	2004	Brazil	GU983196	X
15	2004	Brazil	GU983197	X
59	2005	Brazil	GU983199	X
93	2005	Brazil	GU983200	X
166	2005	Brazil	GU983202	X
26	2009	Brazil	GU983204	X
9	2009	Brazil	GU983205	X
10	2009	Brazil	GU983206	X
3353	2003	China	GQ329725	X

3	2007	China	GQ429279	X
SJ3	2007	China	EU596581	X
SJ5	2007	China	EU596583	X
SJ7	2007	China	EU596584	X
SF6	2007	China	EU596586	X
SF9	2007	China	EU596587	X
13	2008	China	GQ429280	X
20	2008	China	GQ429287	X
4	2010	China	HQ699666	X
12	2010	China	HQ699669	X
26	2010	China	JF742580	X
35	2010	China	JF742586	X
36	2010	China	JF742587	X
3	2010	China	JF742595	X
P1	2010	China	JN788289	X
P6	2010	China	JN788292	X
P11	2010	China	JN788296	X
P15	2010	China	JN788299	X
P17	2010	China	JN788301	X
P19	2010	China	JN788303	X
P29	2010	China	JN788304	X
P41	2010	China	JN788305	X
P45	2010	China	JN788306	X
LY001	2010	China	JQ728980	X
LY003	2010	China	JQ728982	X
QD015	2010	China	JQ728986	X
QD019	2010	China	JQ728989	X
4186	2004	Spain	EU162070	X
4188	2004	Spain	EU162072	X
3961	2004	Spain	EU162073	X
4187	2004	Spain	EU162074	X
4181	2004	Spain	EU162076	X
M123	2007	India	GU477573	X
M121	2007	India	GU477574	X
M110	2007	India	GU477576	X
M125	2007	India	GU477577	X
M120	2007	India	GU477580	X
M140	2007	India	GU477582	X

M133	2007	India	GU477583	X
M114	2007	India	GU477584	X
CJ6	2014	Thailand	KP122024	X
CJ23	2014	Thailand	KP122036	X
CJ48	2014	Thailand	KP122054	X
CJ91	2014	Thailand	KP137045	X
