# Supplementary material for: Phylodynamic Characterization of an Ocular-Tropism Coxsackievirus A24 Variant
Source: PLoS One. 2016 Aug 16;11(8):e0160672. doi: 10.1371/journal.pone.0160672 (PMC4987047; doi:10.1371/journal.pone.0160672)
Supplement: S2 Table — (PDF) [file pone.0160672.s005.pdf]

S2 Table. Best models according to Akaike's information criterion (AICM)

(A) Model composition comparison for the VP 1 region.

Trace	AICM	SE	UCED CON	UCED EXP	UCED LOG	UCED BSP	UCLD CON	UCLD EXP	UCLD LOG	UCLD BSP
UCED CON	10124.33	+/- 0.25	-	821.405	861.431	-8.866	60.364	898.538	917.258	35.16
UCED EXP	10945.74	+/- 0.184	-821.405	-	40.027	-830.271	-761.041	77.133	95.853	-786.245
UCED LOG	10985.77	+/- 0.201	-861.431	-40.027	-	-870.297	-801.068	37.107	55.826	-826.272
UCED BSP	10115.47*	+/- 0.171	8.866	830.271	870.297	-	69.23	907.404	926.124	44.026
UCLD CON	10184.7	+/- 0.265	-60.364	761.041	801.068	-69.23	-	838.174	856.894	-25.204
UCLD EXP	11022.87	+/- 0.296	-898.538	-77.133	-37.107	-907.404	-838.174	-	18.72	-863.378
UCLD LOG	11041.59	+/- 0.177	-917.258	-95.853	-55.826	-926.124	-856.894	-18.72	-	-882.098
UCLD BSP	10159.49	+/- 0.216	-35.16	786.245	826.272	-44.026	25.204	863.378	882.098	-

(B) Model composition comparison for the 3D^{pol} region

Trace	AICM	S.E.	UCED CON	UCED EXP	UCED LOG	UCED BSP	LOG CON	UCLD EXP	UCLD BSP
UCED CON	8594.621	+/- 0.178	-	-26.279	87.036	-46.796	-0.61	-46.7	-46.443
UCED EXP	8568.342	+/- 0.16	26.279	-	113.316	-20.516	25.669	-20.421	-20.164
UCED LOG	8681.657	+/- 0.124	-87.036	-113.316	-	-133.832	-87.647	-133.736	-133.48
UCED BSP	8547.825*	+/- 0.133	46.796	20.516	133.832	-	46.185	0.096	0.352
UCLD CON	8594.011	+/- 0.231	0.61	-25.669	87.647	-46.185	-	-46.089	-45.833
UCLD EXP	8547.921	+/- 0.109	46.7	20.421	133.736	-0.096	46.089	-	0.256

UCLD BSP	8548.178	+/- 0.16	46.443	20.164	133.48	-0.352	45.833	-0.256	-

- SE, standard error; UCED, uncorrelated exponential distribution; UCLD, uncorrelated lognormal distribution; CON, constant; EXP, exponential; LOG, lognormal; BSP, Bayesian skyline plot.
- Models were compared by AICM (SE estimated using bootstrap replicates). Only major estimated parameters with effective sample sizes >200 were compared. The comparisons included one substitution model (SRD06), two relaxed-clock models (UCLD and UCED), and four demographic history models (CON, EXP, LOG, and BSP). For pairwise comparisons, each clock model was combined with one population model. Lower AICM values indicate a better model fit. Differences between AICM estimates are reported. Positive values indicate that models in the rows have a better fit compared to models in the columns. The best model is indicated by an asterisk (*).
