# Supplementary material for: Phylodynamic Characterization of an Ocular-Tropism Coxsackievirus A24 Variant
Source: PLoS One. 2016 Aug 16;11(8):e0160672. doi: 10.1371/journal.pone.0160672 (PMC4987047; doi:10.1371/journal.pone.0160672)
Supplement: S3 Table — (PDF) [file pone.0160672.s006.pdf]

S3 Table. Pairwise comparison of 3D^{pol} nucleotide and amino acid similarity between each CV-A24v genotype and prototype strains of CV-A24 and poliovirus 1–3

Strain name[a]	Similarity[b]	Genotype A	Genotype B	Genotype C	Genotype D	Genotype E
Joseph	Nucleotide	84.6	87.0–88.0	88.4–89.1	87.1–87.7	87.4–88.4
(CV-A24)[c]	Amino acid	98.3	97.8–98.7	98.7–99.1	97.0–98.3	97.8–99.1
Mahoney	Nucleotide	87.3	85.9–86.4	86.8–87.3	86.4–87.3	86.7–87.4
(poliovirus 1)	Amino acid	99.1	97.0–97.8	97.8–98.3	96.1–97.4	97.4–98.3
Lansing	Nucleotide	87.5	87.8–88.3	87.1–87.4	86.2–86.8	85.9–87.5
(poliovirus 2)	Amino acid	100.0	98.7–99.6	99.6–100.0	97.0–98.3	98.3–99.1
Leon	Nucleotide	85.5	86.2–86.7	87.1–88.4	87.5–87.8	88.3–89.0
(poliovirus 3)	Amino acid	98.7	97.8–98.7	98.7–99.1	97.0–98.3	98.3–99.1

a. Strain name (prototype of serotype)

b. Sequence similarities were estimated by p-distance via MEGA7 program.

c. CV-A24: coxsackievirus A24
